# Stress, Diet, and Sleep Shape Irritable-Bowel-Syndrome-Specific Symptoms: The Lockdown “Cocoon Effect”

**DOI:** 10.3390/jcm14238487

**Published:** 2025-11-29

**Authors:** Stefano Kayali, Elisa Marabotto, Giorgia Bodini, Simona Marenco, Sara Labanca, Giulia Pieri, Patrizia Zentilin, Edoardo Giovanni Giannini, Manuele Furnari

**Affiliations:** 1 Department of Medicine and Surgery, University of Parma, 43121 Parma, Italy; 2 Gastroenterology and Endoscopy Unit, University Hospital of Parma, 43126 Parma, Italy; 3 Gastroenterology Unit, Department of Internal Medicine, IRCCS Ospedale Policlinico San Martino, University of Genoa, 16126 Genoa, Italy

**Keywords:** irritable bowel syndrome, environmental factors, lifestyle, management, pathophysiology

## Abstract

**Background/Objectives**: Irritable bowel syndrome (IBS) is a highly prevalent gastrointestinal disorder affecting almost 10% of the general population, characterized by recurrent abdominal pain and altered bowel habits. Its pathophysiology is incompletely understood, but it is established that symptoms result from an interplay between several environmental- and patient-related factors. This study aimed to analyze the influence of a widespread shift in lifestyle habits and multidimensional stress on IBS manifestations. **Methods**: An online survey was administered during the COVID-19 lockdown in 2020 to three groups of people representative of the general population. The survey contained questions regarding socio-demographic data, dietary habits, alcohol, smoking, physical activity, sleeping, working activities, stress level, and the characteristics of gastrointestinal symptoms related to both the pre-pandemic period and the lockdown period. The definition of IBS was based on the Rome IV criteria. Multivariate analyses were used to evaluate the association between environmental variables and the occurrence/resolution of IBS. **Results**: A total of 2735 participants were enrolled. Among them, 122 patients (46.2%) reported symptoms’ improvement during the observation period, while 118 previously healthy subjects (4.8%) developed IBS symptoms. Reduced general stress (OR = 2.2, 95%CI 1.1–4.6, *p* = 0.029), increased fiber intake (OR = 2.8, 95%CI 1.6–5.0, *p* < 0.001), and increased hours of sleep (OR = 2.0, 95%CI 1.1–3.8, *p* = 0.031) were associated with a high probability of IBS resolution, while increased anxiolytic pill intake (OR = 0.14, 95%CI 0.04–0.46, *p* = 0.001) showed a low likelihood of IBS resolution. Reduced physical activity (OR = 2.0, 95%CI 1.3–3.2, *p* = 0.002), increased anti-inflammatory effects (OR = 2.4, 95%CI 1.4–4.1, *p* = 0.002), anxiolytic pill intake (OR = 3.5, 95%CI 2.1–5.9, *p* < 0.001), and increased work-related stress (OR = 1.8, 95%CI 1.2–2.8, *p* = 0.009) were risk factors for IBS symptoms’ occurrence. Reduced alcohol consumption was a protective factor (OR = 0.5, 95%CI 0.3–0.8, *p* = 0.006). The resolution of IBS did not affect upper gastrointestinal functional symptoms (OR = 0.2, 95%CI 0.1–0.3, *p* < 0.001). **Conclusions**: The widespread lifestyle change forced by the pandemic created a protective “Cocoon Effect”, resulting in a beneficial effect in almost half of patients with IBS. Our findings provide large-scale evidence that environmental factors play a pivotal role in the pathophysiology of IBS. Specifically, stress levels, fiber intake, sleep patterns, and alcohol consumption are key modifiable drivers of symptom occurrence and resolution.

## 1. Introduction

Irritable bowel syndrome (IBS) is a highly prevalent gastrointestinal disorder affecting between 5% to 10% of the general population [1]. IBS is responsible for 25 to 50 percent of all referrals to gastroenterologists in industrialized countries [2]. Nevertheless, it is often underrecognized and its prevalence is likely underestimated [3]. This functional disorder is characterized by recurrent abdominal pain and altered bowel habits, with either prevalent diarrhea, constipation, or both [4]. IBS greatly affects patients’ quality of life, both mentally and physically, and is responsible for psychological distress and a severe worsening of quality of life and productivity [5]. Although not life-threatening, it can be responsible for a severe impairment on patients’ quality of life, similar to what is suffered by those affected by other well-known conditions such as diabetes mellitus, dialysis-dependent end-stage renal disease, and depression [6]. It is also the second leading cause of work absenteeism [7]. Due to its high prevalence, its association with redundant medical procedures and drug prescriptions, and its negative effect on work productivity, IBS represents a significant burden on healthcare systems worldwide [5,8,9].

Its pathophysiology is incompletely understood, but it is well-established that symptoms seem to result from an interplay between several environmental- and patient-related factors [10,11]. Among the latter, research has focused on visceral hypersensitivities, altered intestinal permeability, local inflammation, gut dysmotility, dysbiosis, genetic predisposition, and the dysregulation of the gut–brain axis [12]. While the role of these factors is acknowledged, the large-scale, systematic impact of environmental factors remains far more difficult to quantify.

The early part of 2020 saw an unprecedented, societal-wide lifestyle change in Italy caused by the lockdown enforced by the government because of the COVID-19 pandemic [13]. This unique scenario created an inadvertent, large-scale natural experiment, offering a rare opportunity to isolate and measure the impact of complete modifications in lifestyle, daily habits, diet, social interactions, and multidimensional stress on patients with IBS.

Hence, in this study, we aimed to assess the impact of the widespread modification of environmental factors on the course of IBS, including the resolution or new development of its symptoms. We further assessed which environmental factor might have been associated with a higher risk of IBS development (risk factor) or resolution (protective factor) and evaluated whether symptom modification in patients with this disorder affected the onset of upper gastrointestinal functional symptoms.

## 2. Materials and Methods

On 10 April 2020, after 30 days of lockdown, at the apex of the first wave of SARS-CoV-2 in Italy, an online survey was sent via e-mail. The enrolment phase lasted 30 days. Almost 9000 subjects were reached and asked to fill in the electronic survey.

Privacy of the information collected was ensured by password-protected access to the data-containing form. In addition, the anonymous data collected were stored on secured Internet servers in accordance with GDPR (General Data Protection Authority).

Participants were eligible if they were ≥18 years old. Patients already diagnosed with celiac disease or inflammatory bowel diseases were not included since these conditions are characterized by symptoms that can lead to IBS misdiagnosis. To involve a group of participants representative of the general population, with a wide age range and various employment sectors, the survey was disseminated through the mailing lists of three main types of institutions formed by subjects in the juvenile-, work-, and retirement-age demographic: universities, municipalities, and private companies, and associations of pensioners.

The questionnaire dealt with different topics:

- *Subject characteristics and sociodemographic information* [gender, age, height, weight before and after lockdown, location, occupation, type of employment (public, free-lance, retired, student, and unemployed), and work location (working from home, office, and hybrid)].
- *Daily habits and lifestyle before and after lockdown* [physical activity, number of meals per day, amount of food intake, dietary and nutrient composition (water, soft drinks, fruit, fibers, red meat, white meat, fish, and lipids), alcohol consumption, smoking habits, and hours of sleep].
- *Gastrointestinal symptoms* (recurrent abdominal pain, IBS symptoms as categorized by the Rome IV criteria, heartburn, epigastric pain, postprandial fullness, early satiety, nausea, meteorism, tenesmus, and bowel movements per day).
- *Perceived stress dimensions* (general stress, and stress related to work, personal relations, and health status).
- *Medications and dietary supplements* (probiotics, non-steroidal anti-inflammatory drugs (NSAIDs), antacids, sleeping pills, and anxiolytic pills).

The presence and sub-classification of IBS were based on Rome IV criteria [14].

To evaluate any changes in the frequency of IBS symptoms during the lockdown, each of the above variables was examined for any increase, reduction, or stability. These results were then analyzed to determine the association with changes in the course of IBS, including symptom resolution, improvement, or worsening, or new symptom occurrence, both individually and in combination. Once IBS risk and protective factors had been assessed, we investigated whether there was a potential link between IBS modifications and upper gastrointestinal symptoms.

At the end of the analysis of the effect of environmental factor modifications on the course of IBS, we considered the economic burden, including both direct and indirect costs of the disease on the European healthcare systems. Specifically, we applied the resolution rates of IBS observed in our cohort to the estimated population affected by IBS in Italy and Europe. Our aim was to calculate the potential impact of a systematic treatment approach that includes not only pharmaceutical therapies but also environmental factor management on the direct and indirect costs of IBS.

Informed consent regarding personal data management and the research purpose of the questionnaire was collected from every participant in the study. All data were evaluated preserving patients’ anonymity. According to the Italian Medicines Agency det. 20 March 2008 on observational studies using anonymous data, approval by an ethics committee was not mandatory, and informed consent was waived. The study complied with the Declaration of Helsinki (2024 revision).

A Kolmogorov–Smirnov analysis was performed to test the normality of variables. The results of continuous variables were expressed as median and interquartile range. For ordinal and nominal variables, contingency tables were used to indicate the population’s frequency and percentage. Univariate and multivariate Cox method analyses were used to evaluate the association between exposure categorical variables and the occurrence/resolution of IBS. For the non-binary categorical variables, the Wald test was used to obtain the global value of *p*, and Pearson’s chi-squared test was used to determine the association between IBS resolution and the development of upper GI dysfunctional symptoms. A *p*-value < 0.05 was considered statistically significant. Statistical analyses were performed using R, version 4.3.2 software (R Foundation for Statistical Computing).

## 3. Results

From 10 April to May 2020, 2735 participants completed the survey (mean age: 39.7 ± 16.1 years, female gender: 1852, 67.6%). The characteristics of the participants at baseline are illustrated in Table 1. Among them, 264 (9.6%) had symptoms consistent with IBS, while 2471 (90.4%) were not affected by IBS at baseline. Among the former group, during the lockdown, 122 patients (46.2%) reported an improvement in symptoms with a resolution of IBS, while 118 previously negative subjects (4.8%) developed IBS symptoms (*p* < 0.001). Details about the subjects’ characteristics and modifications during the study period are summarized in Supplementary Table S1.

### 3.1. Patients’ Demographic and Social Characteristics

Patients’ characteristics such as age, gender, body mass index, and work-related features (work location, being currently employed, and type of employment) were not associated with a modification in the IBS course. In particular, the previous variables were not statistically significant neither among healthy subjects nor among IBS subjects in the univariate analysis (respectively, age, *p* = 0.67 and *p* = 0.39; BMI, *p* = 0.35 and *p* = 0.93; type of employment, *p* = 0.45 and *p* = 0.59; weight, *p* = 0.58 and *p* = 0.63; gender, *p* = 0.62 and *p* = 0.31; work location, *p* = 0.99 and *p* = 0.89).

### 3.2. Perceived Stress

Among patients with baseline IBS, a reduction in general stress was associated with a greater likelihood of healing (OR = 2.23, 95%CI 1.09–4.56, *p* = 0.029) both in the univariate and multivariate analysis. This significant result refers specifically to general stress. Conversely, when referring to stress subtypes, such as stress related to personal relations, work, and other causes, they are not associated with IBS resolution (*p* = 0.82, *p* = 0.13, *p* = 0.38, respectively). Health-status stress was significant in the univariate analysis (*p* = 0.013) but failed to exhibit any association in the multivariate analysis.

In healthy participants, increased work-related stress was a risk factor for developing IBS symptoms (OR = 1.81, 95%CI 1.16–2.83, *p* = 0.009). Conversely, increased health-status stress was not associated with the onset of the disorder (*p* = 0.79).

The complete multivariate analysis results are shown in Table 2.

### 3.3. Daily Habits and Lifestyle

Modifications in the amount of food intake, number of daily meals, and water reached significance in IBS patients only in the univariate but not in the multivariate analysis (*p* = 0.58, *p* = 0.79, *p* = 0.78). An increase in dietary fiber intake increased the likelihood of IBS symptom resolution (OR = 2.83, 95%CI 1.60–5.02, *p* < 0.001), whereas reducing alcohol consumption lowered the probability of developing IBS symptoms (OR = 0.46, 95%CI 0.26–0.80, *p* = 0.006) (Table 2). Other modifications in dietary habits reached statistical significance neither in patients nor in healthy subjects, in particular, the consumption of fish (*p* = 0.68 and *p* = 0.32, respectively), red meat (*p* = 0.55 and *p* = 0.75), white meat (*p* = 0.48 and *p* = 0.66), lipids (*p* = 0.09 and *p* = 0.07), soft drinks (*p* = 0.83 and *p* = 0.26), fruit (*p* = 0.27 and *p* = 0.33), and water (*p* = 0.78 and *p* = 0.93).

Regarding lifestyle, while reducing athletic activity increased the likelihood of IBS symptom occurrence (OR = 2.04, 95%CI 1.30–3.19, *p* = 0.002), those patients who augmented their sleeping elevated their probability of resolving IBS symptoms (OR = 2.00, 95%CI 1.07–3.76, *p* = 0.031) (Table 2). Smoking habit (either when increased or reduced) showed no statistically significant association with IBS disease course modification neither in patients nor in healthy subjects (*p* = 0.80 and *p* = 0.65, respectively).

### 3.4. Medications and Dietary Supplements

A modification in the number of anxiolytic pills per day was associated with effects on IBS symptoms both in patients and healthy participants. In particular, an increase in their dosage lowered the likelihood of healing symptoms in those already affected (OR = 0.14, 95%CI 0.04–0.46, *p* = 0.001) and increased the probability of a new diagnosis in those healthy at baseline (OR = 3.52, 95%CI 2.08–5.95, *p* < 0.001). An increase in the dosage of anti-inflammatory drugs was a risk factor for the onset of symptoms suggestive of IBS (OR = 2.38, 95%CI 1.39–4.10, *p* = 0.002) (Table 2). The use of probiotics (*p* = 0.20 and *p* = 0.11), antacids (*p* = 0.55 and *p* = 0.15), and sleeping pills (*p* = 0.19 and *p* = 0.16) during the study period was not associated with any effect on the activity of IBS in the multivariate analysis, despite a statistically significant *p* value in the univariate analysis (*p* = 0.01, *p* = 0.05, *p* = 0.02).

**Figure 1:**
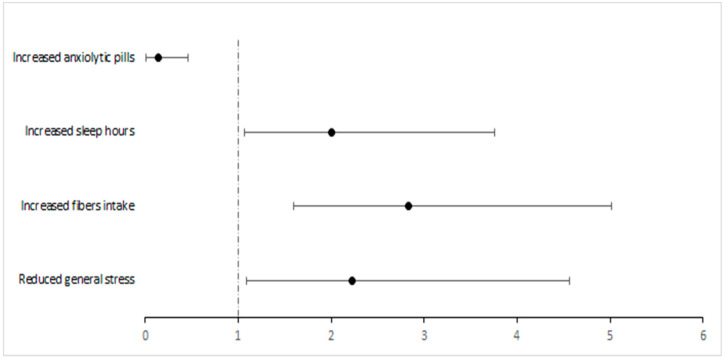
Forest plot of Odds Ratios and 95%CI of protective and risk factors for IBS resolution. The black circles represent the estimated Odds Ratios, the horizontal lines indicate the 95% confidence intervals, and the vertical dashed line marks the null value (OR = 1).

**Figure 2:**
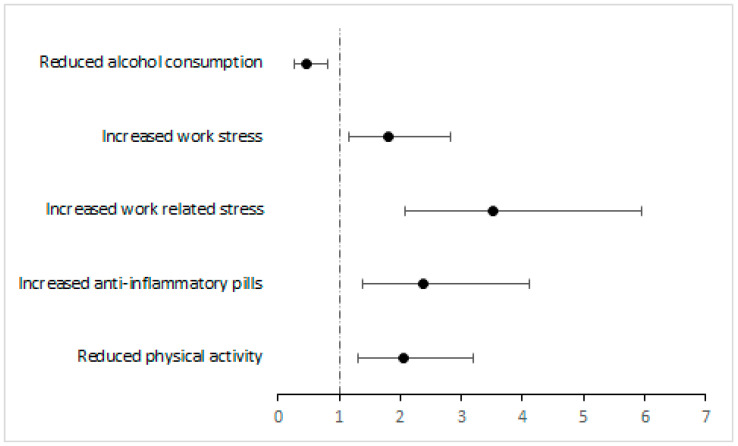
Forest plot of Odds Ratios and 95%CI of protective and risk factors for IBS occurence. The black circles represent the estimated Odds Ratios, the horizontal lines indicate the 95% confidence intervals, and the vertical dashed line marks the null value (OR = 1).

The statistically significant protective and risk factors for IBS development and resolution are summarized in Figure 1 and Figure 2.

Finally, the resolution of IBS did not affect the onset of upper gastrointestinal functional symptoms (OR = 0.18, 95%CI 0.11–0.31, *p* < 0.001).

## 4. Discussion

Our study describes the impact of systematic environmental modification on self-referred symptoms in patients with IBS, leveraging a unique, large-scale natural experiment.

The overall prevalence of IBS in the respondents to our questionnaire was approximately 10%, four-fold higher in women than in men (81.8% vs. 18.2%). These results align with the epidemiological evidence of the literature, as, in Italy, the prevalence of IBS in the general population is estimated between 5.4% and 11.5%. Thus, we feel that these results confirm that the population included in our study is representative of the general population in our country [15].

In our survey, we observed that the environmental factors associated with modifications in the course of the disease could be grouped into four main classes related to the following: stress, dietary habits, physical activity, and the sleep–wake cycle.

As far as stress is concerned, the effect of stressful factors on IBS, both for the disease onset and worsening of symptoms in patients with a pre-existing diagnosis, is well-reported in the literature [5,16]. In our study, it became clear that most individuals, both healthy or suffering from IBS, went in a comparable manner to meet an increase in perceived stress (55.8% vs. 57.8%). This phenomenon, however, has had different consequences in the two groups of people. More specifically, among healthy subjects, work-related stress is a clear risk factor for IBS symptoms since it was statistically associated with an almost double risk (OR = 1.81, *p* = 0.009) of developing the disorder. This result aligns with what has already been reported in the literature [17]. To date, few studies have investigated the relationship between occupational factors and irritable bowel syndrome and the evidence is scarce [18,19,20]. In particular, our data are in line with the study by Huerta-Franco et al. and with the recent meta-analysis by Arif et al. who underlined the detrimental effect of occupational stress on chronic gastrointestinal diseases [19,21]. These findings are also consistent with recent evidence showing that stress, anxiety, and depression were major mediators of symptom exacerbation and new-onset IBS during lockdown periods [22,23]. Social isolation, the disruption of daily routines, and limited access to healthcare and preferred foods were all identified as triggers for symptom deterioration [24].

The detrimental effect of stress in IBS is also supported by how the symptoms are modified when stressful factors decrease. More specifically, patients who have reduced perceived stress (36.7%) were found to have twice the likelihood (OR = 2.23, *p* = 0.029) of healing. Therefore, in our study, stressful factors seemed to be positively associated with the onset of IBS symptoms. Their increase was shown to be a risk factor for the occurrence of the disease, while their reduction showed a helpful effect on the resolution of IBS symptoms.

The use of anxiolytic drugs, by nature related to higher levels of stress and anxiety, has shown a statistically significant association with modifications in the disease course both in healthy and in affected participants. A small proportion of subjects, 10.2% of patients and 6.5% of healthy individuals, reported an increase in the intake of anxiolytics. In affected subjects, augmented consumption was associated with a lower chance of recovery (OR = 0.14 *p* = 0.001). At the same time, in healthy individuals, the risk of developing the disease was found to be three times greater (OR = 3.52, *p* < 0.001). Therefore, our findings support the link between psycho-social factors and IBS symptoms. Unfortunately, the direction of causality in this report cannot be determined since the present study has a cross-sectional drawing.

Nevertheless, it is reasonable that the anxiolytic drug intake was increased because it was already not helpful in the IBS course. The modifications of other environmental factors that happened during the lockdown were shown to be ineffective in this group of subjects as well. In particular, as has already been demonstrated in the literature [25], it is reasonable that those who increased their intake of anxiolytics were significantly less prone to improve their attitude to follow healthier habits in terms of diet and physical activity. Thus, it is reasonable to assume that patients affected by IBS with altered psycho-social factors should be considered as a specific subpopulation of patients in which the improvement in the psychologic sphere takes a fundamental role in the management of the disease activity. In parallel, our finding that the increased intake of anti-inflammatory drugs was associated with a more-than-two-fold higher risk of developing IBS-specific symptoms is biologically plausible: NSAIDs are well known to increase intestinal permeability and induce small-bowel mucosal injury, thereby promoting low-grade inflammation and visceral hypersensitivity, mechanisms consistently reported in IBS pathophysiology [26].

Dietary habits have faced several modifications during the lockdown. According to Scarmozzino et al., about 50% of the Italian population has changed its diet [27]. More specifically, they have shown that there has been greater use of “comfort foods”, such as meals rich in carbohydrates and fats [27]. It is also known in the literature that an unbalanced diet can influence the symptoms of patients suffering from IBS [28,29]. Nevertheless, in our study, the increase in these types of foods is not associated with a significant risk of disease development. Changes in the intake of fibers and vegetables, classically related to a balanced Mediterranean diet, have been proven to be able to influence the IBS symptoms in patients. In particular, the increase in the consumption of fibers was associated with an almost triple probability of resolution of IBS symptoms (OR = 2.83, *p* < 0.001). In the literature, the focus has been recently put on the role of fibers in IBS. There is accumulating evidence that dietary fibers have a “prebiotic role”, able to positively influence the intestinal microbiota composition and local immune response [30,31]. Upon the fermentation of dietary fibers, the resulting short-chain fatty acids (acetate, propionate, and butyrate) seem to improve mucosal permeability and also influence the neuroendocrine system (NES) of the gastrointestinal tract and, consequently, its secretion and motility [32].

With regard to alcohol consumption, its reduction has shown a protective effect (OR = 0.46, *p* = 0.006) on the development of symptoms consistent with IBS. This finding is consistent with the existing literature and is in line with the current American Gastroenterological Association (AGA) dietary recommendations, which advise patients with IBS to reduce their alcohol intake as part of general lifestyle and dietary management [33,34].

The observed lifestyle shifts caused by pandemic restrictive measures also resulted in changes in physical activity and daily hours of sleep.

Most subjects have increased their daily hours of sleep comparably in both groups (40.3% vs. 42.8%, healthy and patients, respectively). Such a change has shown statistically significant effects in the patients’ group, as a longer sleep duration was associated with a two-fold likelihood of IBS symptom resolution (OR = 2.00, *p* = 0.031). These findings are consistent with recent evidence demonstrating a bidirectional relationship between sleep quality and IBS symptoms [35]. Disrupted sleep can heighten visceral pain sensitivity and exacerbate the overall symptom burden, while IBS symptoms themselves further impair sleep, sustaining a self-perpetuating cycle [36].

Physical activity decreased comparably in both groups (40.9% vs. 38.6% in healthy patients). In particular, in healthy subjects, this reduction was associated with a two-fold more significant risk of developing symptoms consistent with irritable bowel syndrome (OR = 2.04, *p* = 0.002), in line with the previous literature [37]. Recent studies have further shown that a sedentary lifestyle is independently associated with both an increased risk of IBS and greater symptom severity, with Odds Ratios ranging from 1.1 to 4.4 across large population-based cohorts [38]. Mechanistically, sedentary behavior is thought to exacerbate IBS through impaired gut motility, enhanced visceral hypersensitivity, the adverse modulation of the gut microbiome, and the amplification of stress-related gut–brain axis dysfunction [39]. Our results support the importance of the recently suggested specific management and prescription of physical activity in patients with IBS [40,41]. These notwithstanding, it seems that an improved approach to physical activity should not be suggested alone but together with the optimization of dietary habits and psychosocial factors.

A surprising finding emerged from our data: during this period of deep change in environmental factors, a clinical resolution of symptoms occurred in almost half of the cases (46.2%). On the other hand, in healthy subjects, the percentage that developed IBS has only settled to 4.8% (*p* < 0.001). While other studies have termed this the ‘COVID-19-IBS Paradox’ [42,43,44], our findings allow us to propose a mechanistic explanation: the ‘Cocoon Effect’. The key components of this protective effect appear to be multifactorial, including the better management of symptoms at home rather than at work or in public places. Free access to toilets without social or work constraints may allow patients to better withstand recurrent abdominal pain and urgency, disease characteristics that severely impair quality of life [45]. The forced home stay also allowed for increasing the hours of sleep every day, the latter being a factor able to influence the disease course in patients positively. Finally, the more regular timings of meals and the slightest intake of refined foods, typically consumed at work, may have contributed to this effect.

These observations align with recent reports showing that some IBS patients experienced symptom improvement during lockdowns, possibly due to the reduced exposure to work stressors, improved sleep, and more regular meal patterns, even amid widespread psychosocial distress [43]. Although the lockdown represented an exceptional context, some elements of the “Cocoon Effect” may still hold relevance in routine life. Reduced work-related stress, greater flexibility in daily routines, and easier access to restrooms could be partially replicated outside a lockdown scenario, and remote or hybrid working arrangements for selected patients might help recreate some of the protective conditions observed during this period. Furthermore, the positive effects observed may even underestimate the true potential of environmental modulation in IBS, given the stressful context related to the increase in stress perceived by patients during the pandemic.

IBS is a chronic condition that severely impairs the patient’s quality of life. Currently, the predominant approach to moderate to severe disease activity is pharmacological. However, only a few randomized controlled trials have investigated the use of these drugs in combination with other supportive therapies, their optimal duration, or whether they should be administered continuously or as needed [46]. In any case, a selective, exclusive pharmaceutical approach is often unsuccessful for an extended period.

Our study provides a strong rationale for reinforcing a systematic evaluation of environmental factors into the standard of care for all patients with IBS. Since no medical therapy is proven to alter the natural history of the disease, our findings reinforce the importance of analyzing environmental factors and adopting a patient-tailored approach to achieve the best clinical outcomes.

Based on our survey, we suggest drawing up a simple questionnaire to be filled out by patients. This could assist both patients and physicians in determining the factors that most affect IBS disease activity, leading to an integrated approach involving specific drugs and lifestyle, dietary, and environmental modifications, guided by medical advice and increased self-awareness. Future studies will be necessary in order to develop the abovementioned questionnaire based on our results, followed by works to validate it.

Irritable bowel syndrome is the leading cause of referral to gastroenterological care in industrialized countries [47,48,49] and, according to Flacco et al., one of the costliest chronic disorders, with an annual per-patient burden comparable to or even exceeding that of asthma, chronic obstructive pulmonary disease, or diabetes [50,51,52,53]. It is therefore reasonable that acting on the environmental factors highlighted in our study could have major implications not only for patient well-being but also for the sustainability of healthcare systems.

The study has both strengths and limitations. A significant strength of this study is the large number of participants, including different age groups and social backgrounds, representative of the general population; with 2735 subjects, this study has analyzed the largest number of cases among similar studies in the literature. Furthermore, all of the few studies published until now have focused on specific and limited environmental factors capable of modifying symptoms of irritable bowel syndrome. In our work, we have analyzed, individually and overall, in an integrated approach, numerous environmental factors to decrease any bias linked to restricted analyses to only some issues, not representative of reality.

The study presents limitations that should be acknowledged. The end of the enrolment phase was set at 60 days after the beginning of the lockdown in Italy and has allowed us to evaluate the effects of the first two month of restrictive measures. However, the pandemic restrictive measures continued for several other weeks for different working classes. Therefore, it is not possible to exclude the possibility that the distribution of IBS symptoms might differ after this period. As a result, both the resolution and development rates of IBS might have been underestimated. Speaking of the percentage of the new onset of IBS-like symptoms, it is important to underline how the result of 4.8% could be both under- or overestimated since those symptoms were present for not more than 60 days. Since at least six months are requested by the Rome IV criteria, part of that 4.8% might be due to acute causes of abdominal symptoms rather than being truly IBS related. Furthermore, the duration of the forced confinement of only 60 days before the survey administration could be considered insufficient for verifying some effects. However, a previous review by Brooks et al. showed how a duration of only ten days is already able to determine relevant psycho-social consequences [54]. A further limitation was represented by sending the questionnaire via the web; it might have resulted in excluding participants without Internet access. Finally, the survey mode did not let us exclude eventual organic causes of recurrent abdominal pain and diarrhea, such as celiac disease (CD), inflammatory bowel diseases (IBD), and microscopic colitis (MC). In our opinion, this is a partial limitation since we did not enroll people with known CD, IBD, or MC, as stated in the exclusion criteria. Furthermore, the latest American College of Gastroenterology guidelines support our positive diagnostic strategy for IBS, and a study evaluating the overlap between IBS, IBD, and MC found that fewer than two percent of IBS patients actually had IBD or MC [55,56]. With regard to celiac disease, even if a negative serology is needed to diagnose IBS, it is unlikely that, among the 46.2% of patients healed during lockdown, a considerable part was formed by undiagnosed CD patients who improved without specific therapy.

## 5. Conclusions

This study reinforces the pivotal role of environmental factors in IBS, showing that a widespread lifestyle shift can induce clinical remission in nearly half of affected individuals. We propose the “Cocoon Effect” as a potential explanation for this phenomenon, that is, a protective personal environment where patients can better regulate diet, sleep, and stress. Our findings strengthen the view that lifestyle modification is a core component of IBS management, supporting its systematic and structured integration within an individualized care model. Pharmacological therapy should be combined with interventions targeting stress, dietary habits, alcohol consumption, sleep–wake rhythms, and physical activity. Adopting such a comprehensive approach has the potential not only to substantially improve patient quality of life but also to reduce the economic burden of IBS on healthcare systems.

## Figures and Tables

**Table 1:** Subjects’ characteristics at baseline according to IBS status pre-lockdown.

Variables	Colonna1	Healthy Subjects Pre-Lockdown	IBS Subjects Pre-Lockdown	*p* Value
	2735	2471	264	
Gender (%)	Female	1635 (66.2)	216 (81.8)	<0.001
Age (median [IQR])		37.00 [24.00, 56.00]	34.00 [23.00, 55.00]	0.168
Height (median [IQR])		168 [162, 175]	165.00 [160.00, 171.00]	<0.001
BMI (median [IQR])		22.59 [20.31, 25.25]	22.22 [20.20, 24.91]	0.124
BMI (%)	Regular	1637 (66.2)	173 (65.5)	0.117
	Obese class I	106 (4.3)	8 (3.0)	
	Obese class II	24 (1.0)	0 (0.0)	
	Obese class III	7 (0.3)	2 (0.8)	
	Overweight	523 (21.2)	54 (20.5)	
	Underweight	174 (7.0)	27 (10.2)	
Employment (%)	Freelance	150 (6.1)	14 (5.3)	0.139
	Private employee	137 (5.5)	8 (3.0)	
	Public employee	179 (47.7)	113 (42.8)	
	Retired	48 (1.9)	3 (1.1)	
	Student	917 (37.1)	122 (46.2)	
	Unemployed	40 (1.6)	4 (1.5)	

**Table 2:** Risk and protective factors for IBS development and resolution. ^‡^ Cox multivariate analysis.

Effect	Variable	OR (95%CI)	*p* ^‡^
IBS resolution	Reduced general stress	2.23 (1.09–4.56)	**0.029**
	Increased general stress	0.78 (0.41–1.45)	0.43
	Increased fiber intake	2.83 (1.60–5.02)	**<0.001**
	Reduced fiber intake	1.22 (0.45–3.27)	0.68
	Increased sleep hours	2.00 (1.07–3.76)	**0.031**
	Reduced sleep hours	1.22 (0.59–2.48)	0.59
	Increased anxiolytic pills	0.14 (0.04–0.46)	**0.001**
	Reduced anxiolytic pills	4.66 (0.50–43.3)	0.18
IBS development	Reduced physical activity	2.04 (1.30–3.19)	**0.002**
	Increased physical activity	0.81 (0.42–1.55)	0.52
	Reduced alcohol consumption	0.46 (0.26–0.80)	**0.006**
	Increased alcohol consumption	0.67 (0.35–1.27)	0.22
	Increased anti-inflammatory pills	2.38 (1.39–4.10)	**0.002**
	Reduced anti-inflammatory pills	0.94 (0.47–1.88)	0.87
	Increased anxiolytic pills	3.52 (2.08–5.95)	**<0.001**
	Reduced anxiolytic pills	1.68 (0.55–5.01)	0.36
	Increased work-related stress	1.81 (1.16–2.83)	**0.009**
	Reduced work-related stress	1.13 (0.67–1.89)	0.65
Onset of upper GI functional symptoms	IBS resolution	0.18 (0.11–0.31)	**0.001**

## Data Availability

The data are available upon request.

## Supplementary Materials

The following supporting information can be downloaded at: https://www.mdpi.com/article/10.3390/jcm14238487/s1. Table S1. Patients’ characteristics and their modifications during lockdown.

## Abbreviations

The following abbreviations are used in this manuscript:

IBS	Irritable Bowel Syndrome
MC	Microscopic Colitis
CD	Celiac Disease
BMI	Body Mass Index
NSAIDs	Non-Steroidal Anti Inflammatory Drugs
