# Supplementary material for: Stress, Diet, and Sleep Shape Irritable-Bowel-Syndrome-Specific Symptoms: The Lockdown “Cocoon Effect”
Source: J Clin Med. 2025 Nov 29;14(23):8487. doi: 10.3390/jcm14238487 (PMC12693210; doi:10.3390/jcm14238487)
Supplement: Supplementary file 1 [file jcm-14-08487-s001.zip › jcm-4012146-supplementary.pdf]

Supplementary Material, Table S1. Patients' characteristics and their modifications during lockdown

Variables' topics	Variables		Healthy subjects pre-lockdown	IBS subjects pre-lockdown
	n	2735	2471	264
Daily habits and lifestyle	Alcohol consumption (%)	Discontinued	290 (11.7)	35 (13.3)
		Increased	295 (11.9)	27 (10.2)
		Much reduced	397 (16.1)	38 (14.4)
		Reduced	270 (10.9)	25 (9.5)
		Unchanged	1219 (49.3)	139 (52.7)
	Physical activity (%)	Increased	489 (19.8)	54 (20.5)
		Reduced	1010 (40.9)	101 (38.3)
		Unchanged	972 (39.3)	109 (41.3)
	Hours of sleep (%)	Increased	995 (40.3)	113 (42.8)
		Reduced	512 (20.7)	71 (26.9)
		Unchanged	964 (39.0)	80 (30.3)
	Work location (%)	Dismissed	390 (15.8)	58 (22.0)
		Hybrid	29 (1.2)	3 (1.1)
		Smart working	1681 (68.0)	178 (67.4)
		Unchanged	371 (15.0)	25 (9.5)
	Daily meals (%)	Increased	497 (20.1)	58 (22.0)
		Reduced	311 (12.6)	54 (20.5)
		Unchanged	1663 (67.3)	152 (57.6)
	Lipids (%)	Increased	589 (23.8)	70 (26.5)
		Reduced	613 (24.8)	73 (27.7)
		Unchanged	1269 (51.4)	121 (45.8)
	Fish (%)	Increased	488 (19.7)	56 (21.2)
		Reduced	517 (20.9)	65 (24.6)
		Unchanged	1466 (59.3)	143 (54.2)
	Food amount (%)	Increased	829 (33.5)	97 (36.7)
		Reduced	514 (20.8)	64 (24.2)
		Unchanged	1128 (45.6)	103 (39.0)
	Fruit (%)	Increased	888 (35.9)	99 (37.5)

		Reduced	217 (8.8)	37 (14.0)
		Unchanged	1366 (55.3)	128 (48.5)
	Soft drinks (%)	Increased	258 (10.4)	30 (11.4)
		Reduced	819 (33.1)	105 (39.8)
		Unchanged	1394 (56.4)	129 (48.9)
	Red meat (%)	Increased	319 (12.9)	35 (13.3)
		Reduced	519 (21.0)	60 (22.7)
		Unchanged	1633 (66.1)	169 (64.0)
	Fibres (%)	Increased	900 (36.4)	99 (37.5)
		Reduced	173 (7.0)	23 (8.7)
		Unchanged	1398 (56.6)	142 (53.8)
	Water (%)	Increased	651 (26.3)	89 (33.7)
		Reduced	378 (15.3)	39 (14.8)
		Unchanged	1442 (58.4)	136 (51.5)
	Smoke (%)	Discontinued	31 (1.3)	3 (1.1)
		Increased	183 (7.4)	26 (9.8)
		Reduced	99 (4.0)	16 (6.1)
		Unchanged	2158 (87.3)	219 (83.0)
Perceived stress	Health status stress (%)	Increased	798 (32.3)	73 (27.7)
		Reduced	303 (12.3)	52 (19.7)
		Unchanged	1370 (55.4)	139 (52.7)
	Other stress (%)	Increased	445 (18.0)	59 (22.3)
		Reduced	300 (12.1)	37 (14.0)
		Unchanged	1726 (69.9)	168 (63.6)
	Personal relations stress (%)	Increased	749 (30.3)	86 (32.6)
		Reduced	466 (18.9)	63 (23.9)
		Unchanged	1256 (50.8)	115 (43.6)
	Stress general (%)	Increased	1378 (55.8)	134 (50.8)
		Reduced	626 (25.3)	97 (36.7)
		Unchanged	467 (18.9)	33 (12.5)
	Work stress (%)	Increased	639 (25.9)	57 (21.6)
		Reduced	594 (24.0)	76 (28.8)

		Unchanged	1238 (50.1)	131 (49.6)
Medications and medical care	Antacids (%)	Increased	147 (5.9)	32 (12.1)
		Reduced	104 (4.2)	29 (11.0)
		Unchanged	2220 (89.8)	203 (76.9)
	Anti-inflammatory pills (%)	Increased	170 (6.9)	23 (8.7)
		Reduced	268 (10.8)	43 (16.3)
		Unchanged	2033 (82.3)	198 (75.0)
	Anxiolytic pills (%)	Increased	160 (6.5)	27 (10.2)
		Reduced	59 (2.4)	6 (2.3)
		Unchanged	2252 (91.1)	231 (87.5)
	Probiotics (%)	Increased	214 (8.7)	37 (14.0)
		Reduced	160 (6.5)	43 (16.3)
		Unchanged	2097 (84.9)	184 (69.7)
	Sleeping pills (%)	Increased	185 (7.5)	32 (12.1)
		Reduced	64 (2.6)	12 (4.5)
		Unchanged	2222 (89.9)	220 (83.3)
Gastrointestinal symptoms	Chronic symptoms severity (%)	Increased	283 (11.5)	59 (22.3)
		Reduced	345 (14.0)	47 (17.8)
		Unchanged	1843 (74.6)	158 (59.8)
	Daily bowel movements (%)	Increased	445 (18.0)	42 (15.9)
		Reduced	192 (7.8)	70 (26.5)
		Unchanged	1834 (74.2)	152 (57.6)
	Early satiety (%)	Improved	22 (0.9)	7 (2.7)
		Unchanged	2259 (91.4)	212 (80.3)
		Worsened or new onset	190 (7.7)	45 (17.0)
	Epigastric pain (%)	Improved	41 (1.7)	21 (8.0)
		Unchanged	2159 (87.4)	186 (70.5)
		Worsened or new onset	271 (11.0)	57 (21.6)
	Heartburn (%)	Improved	35 (1.4)	7 (2.7)
		Unchanged	2262 (91.5)	212 (80.3)
		Worsened or new onset	174 (7.0)	45 (17.0)
	Meteorism (%)	Improved	52 (2.1)	27 (10.2)

		Unchanged	2044 (82.7)	152 (57.6)
		Worsened or new onset	375 (15.2)	85 (32.2)
	Nausea (%)	Improved	23 (0.9)	9 (3.4)
		Unchanged	2197 (88.9)	180 (68.2)
		Worsened or new onset	251 (10.2)	75 (28.4)
	Postprandial fullness (%)	Improved	46 (1.9)	25 (9.5)
		Unchanged	2071 (83.8)	157 (59.5)
		Worsened or new onset	354 (14.3)	82 (31.1)
	Symptoms frequency (%)	Every day	146 (5.9)	54 (20.5)
		Few times per month	240 (9.7)	48 (18.2)
		Few times per week	305 (12.3)	77 (29.2)
		Several times per week	143 (5.8)	37 (14.0)
		Never	1637 (66.2)	48 (18.2)
	Symptoms timing (%)	Both	149 (6.0)	52 (19.7)
		Daytime	601 (24.3)	147 (55.7)
		Never	1637 (66.2)	48 (18.2)
		Night time	84 (3.4)	17 (6.4)
	Tenesmus (%)	Improved	15 (0.6)	11 (4.2)
		Unchanged	2311 (93.5)	205 (77.7)
		Worsened or new onset	145 (5.9)	48 (18.2)
